# Deletion of resistin-like molecule-beta attenuates angiotensin II-induced abdominal aortic aneurysm

**DOI:** 10.18632/oncotarget.22042

**Published:** 2017-10-24

**Authors:** Xiao Meng, Kai Zhang, Jing Kong, Long Xu, Guipeng An, Weidong Qin, Jifu Li, Yun Zhang

**Affiliations:** ^1^ The Key Laboratory of Cardiovascular Remodeling and Function Research, Chinese Ministry of Education and Chinese Ministry of Health, Qilu Hospital, Shandong University, Jinan, 250012, China

**Keywords:** abdominal aortic aneurysm, RELMβ, inflammation, matrix metalloproteinase

## Abstract

In the present study, we want to test whether deletion of resistin-like molecule-beta (RELMβ) attenuates angiotensin II (Ang II)-induced formation of abdominal aortic aneurysm (AAA). RELMβ gene expression was inhibited by siRNA both *in vivo* and *in vitro*. Apolipoprotein E-knockout (ApoE^−/−^) mice were randomly divided into saline, Ang II, siRNA negative control (si-NC) and siRNA RELMβ (si-RELMβ) groups (n=15 each), and mice in the last three groups underwent Ang II infusion for 4 weeks to induce AAA. RELMβ gene deficiency significantly decreased AAA incidence and severity, which was associated with reduced macrophage accumulation and decreased expression of proinflammatory cytokines (monocyte chemoattractant protein 1 and interleukin 6), matrix metalloproteinase 2 (MMP-2) and MMP-9 in the aortic wall. In cultured macrophages, RELMβ deficiency blunted the response of macrophages to Ang II and downregulated the levels of proinflammatory cytokines, MMP-2 and MMP-9. Recombinant RELMβ promoted the secretion of proinflammatory cytokines, MMP-2 and MMP-9 in macrophages and activated extracellular signal-regulated kinase 1/2 (ERK1/2) and c-Jun N-terminal kinase (JNK) signaling, which was reversed with pretreatment with inhibitors of ERK1/2 and JNK. Deletion of RELMβ attenuated Ang II-induced AAA formation in ApoE^−/−^ mice. The inherent mechanism may involve the reduced expression of proinflammatory cytokines, MMP-2 and MMP-9, which was mediated by ERK1/2 and JNK activation. Therefore, inhibiting RELMβ secretion may be a novel approach for anti-aneurysm treatment.

## INTRODUCTION

Abdominal aortic aneurysm (AAA) is a structural deterioration of tissue architecture leading to progressively enlarged abdominal aorta. It represents high mortality with rupture in the adult population and is an increasing health concern in the whole world [1]. The pathophysiological mechanism underling this disease remains unclear and therapeutic options are limited. Despite surgical advantage in patients with large AAAs, current effective therapeutic strategies for treating patients in whom surgery is not indicated (asymptomatic, small or no surgery indicated) or contraindications are lacking and are needed.

Resistin is an adipocyte-secreted factor that leads to insulin resistance [2]. Three resistin-like molecules (RELMs), RELMα, RELMβ, and RELMγ, have been identified. RELMs have similar biological activities to resistin and regulate insulin sensitivity. Unlike RELMα and RELMγ, which are found only in mice, RELMβ is also present in human tissues. Initially, RELMβ was considered strictly limited to the gastrointestinal tract, especially colon. Recent evidence indicated that RELMβ was also present in blood, lung, heart, kidney and adrenal gland and notably expressed in macrophages, foam cells, T cells and smooth muscle cells [3-6]. Therefore, RELMβ has received increasing attention. Studies found that it contributed to immune response regulation and was involved in the pathogenesis of many human diseases, such as asthma and pulmonary hypertension [7, 8]. However, the precise physiological function of RELMβ has not been fully elucidated.

Clinical evidence suggests that AAA formation shares some common features with coronary atherosclerosis disease, and AAA has been considered the risk equivalent of coronary atherosclerosis disease. One recent study revealed the expression of RELMβ in foam cells of atherosclerotic plaques and its effect on promoting the development of atherosclerosis [6]. Inspired by the finding of an association between RELMβ and atherosclerosis, we wondered whether RELMβ expression was associated with AAA progression. We designed a primary experiment and found enhanced RELMβ mRNA and protein expression in aortic tissues of AAA mouse models than those in control mice [9]. In addition, the mRNA and protein expression of RELMβ was greater in human aneurysmal aortas than non-aneurysmal ones [9]. Moreover, serum RELMβ level assessed by ELISA was significantly greater in AAA patients than non-aneurysmal patients.

Therefore, we hypothesized that increased expression of RELMβ was actually related to the formation of AAA. In the present study, we provide results of direct studies including a series of ***in vivo*** and ***in vitro*** experiments to test this hypothesis and further to delineate the exact mechanism of RELMβ in the AAA progression.

## RESULTS

### Transfection efficiency of RELMβ siRNA *in vivo* and *in vitro*

The siRNA technology could effectively suppress the target gene expression and has been used for experimental and therapeutic purposes [12]. The siRNA targeting RELMβ was transfected into mice and macrophages for gene silencing. Transfection efficiency of RELMβ siRNA was assessed *in vivo* and *in vitro*. As expected, the RELMβ mRNA and protein expression in suprarenal aortic tissue was significantly decreased after transfection. Similar results were shown in *in vitro* study (Supplementary Figure 1).

### Deletion of RELMβ gene reduced the incidence and severity of Ang II-induced AAA in mice

**Figure 1:**
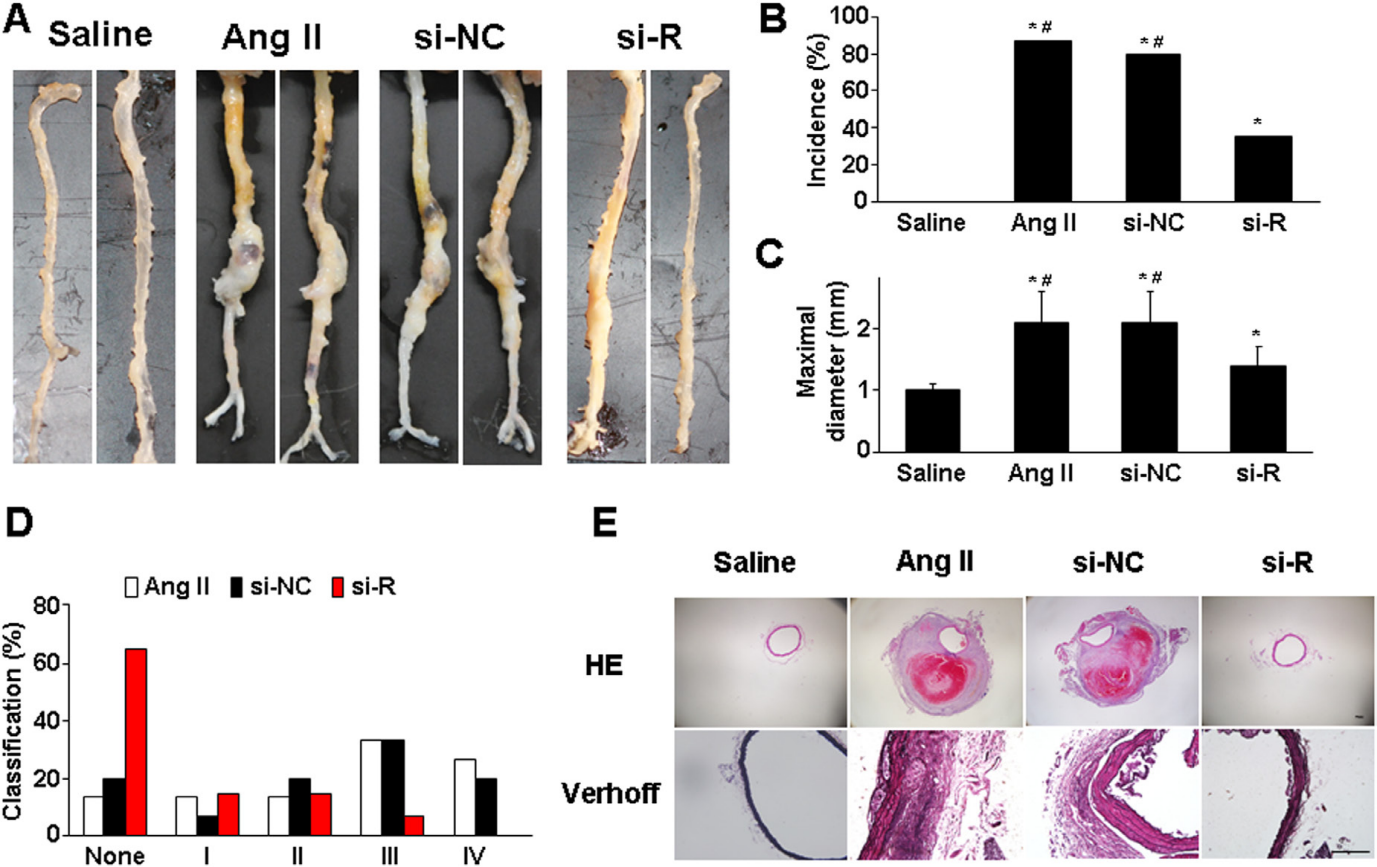
Effect of RELMβ gene deletion on the development of AAA and aortic wall composition in ApoE^−/−^ mice **(A)**, Representative abdominal aortic specimens in 4 groups of mice; **(B)**, Incidence of AAA; **(C)**, Maximal diameters of suprarenal aortas; **(D)**, Classification of AAA; **(E)**, Representative H&E and Verhoff staining. si-NC, si-negative control; si-R, si-RELMβ. ^*^P <0.05 vs. saline group, #*P* <0.05 vs. si-R group. Scale bar: 100 μm.

Ang II infusion for 4 weeks could induce AAA formation in ApoE^−/−^ mice, and saline infusion produces no aneurysm [13, 14]. In this study, the incidence of aortic aneurysm was 86.7% (13/15) with Ang II induction, but was significantly decreased with si-RELMβ treatment (35.7%, 5/14) (*P* <0.05, Figure 1A and 1B). RELMβ deficiency reversed the increase of aortic diameter induced by Ang II as compared with Ang II alone and si-NC treatment (*P* <0.05, Figure 1C). Moreover, si-RELMβ treatment produced type I or II forms of aneurysm severity, which was relatively less than that with Ang II alone and si-NC treatment (most with type III or IV forms) (Figure 1D). Therefore, RELMβ deficiency attenuated the incidence and severity of Ang II-stimulated AAA formation in mice.

### Systolic blood pressure (SBP) and lipid profiles of mice

Exogenous Ang II infusion for 4 weeks could significantly elevate SBP in ApoE^−/−^ mice [14]. In this study, RELMβ knockdown had no effect on Ang II-induced SBP (Supplementary Table 1). Meanwhile, the serum lipid levels did not differ among the 3 groups of mice, which suggested that RELMβ did not affect lipid metabolism of mice (Supplementary Table 2).

### RELMβ knockdown improved aortic wall remodeling

Ang II infusion remodels the abdominal aortic wall, including a thickened aortic wall, breakdown of media and adventitia, disruption of intima with thrombus formation, and discontinuity of elastin fibers [14, 15]. H&E and VVG staining revealed dilated aorta, luminal thrombosis and disrupted medial elastin in Ang II and si-NC groups (Figure 1E). However, RELMβ knockdown lessened these pathological changes, and markedly improved the aortic histology.

### RELMβ knockdown attenuated macrophage infiltration and the inflammatory response

**Figure 2:**
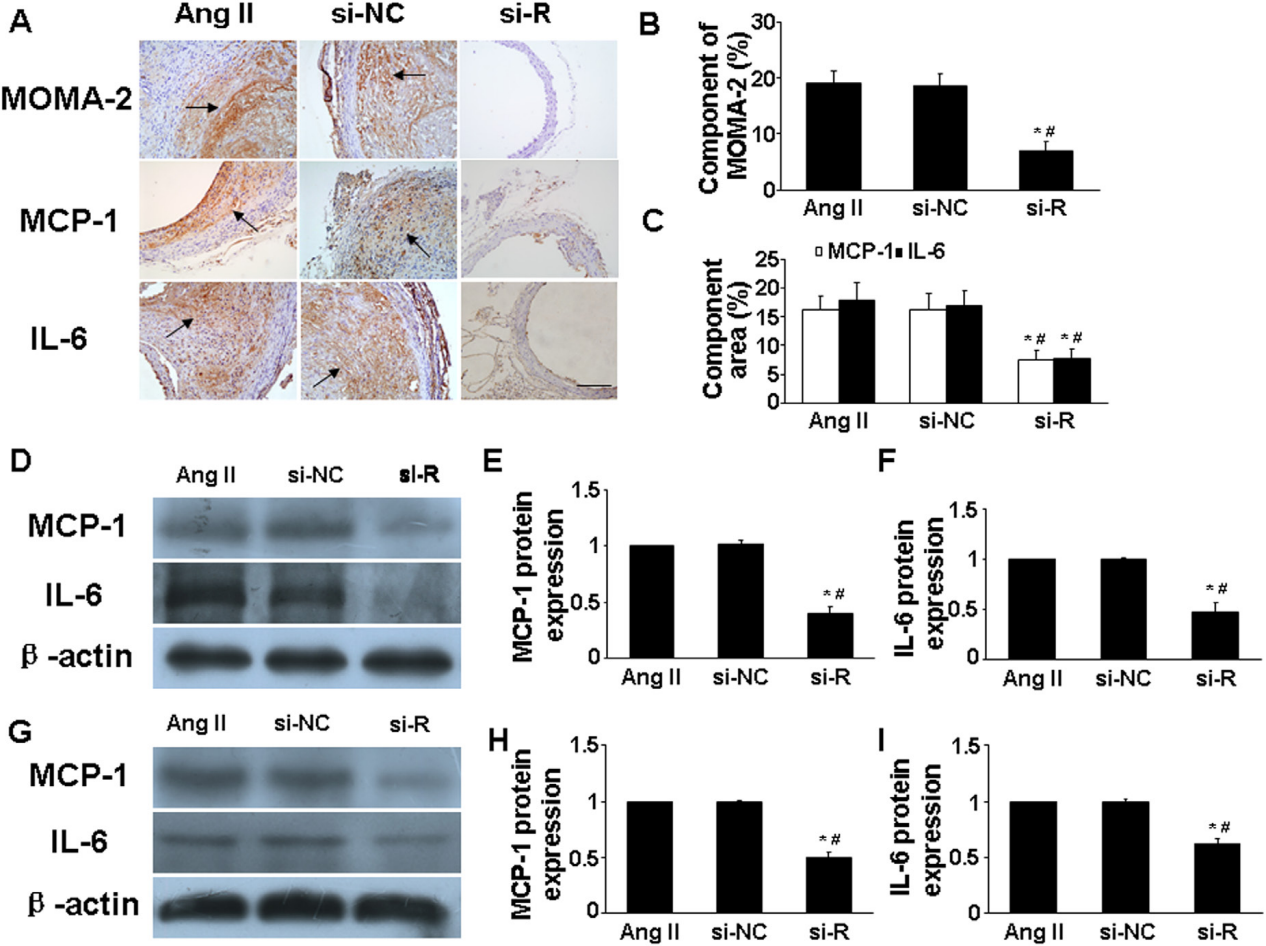
Effect of RELMβ gene deletion on the infiltration of proinflammatory cells and expression levels of proinflammatory cytokines *in vivo* and *in vitro* **(A)**, Representative MOMA-2, MCP-1 and IL-6 staining in 3 groups of mice; Scale bar: 100 μm. **(B** and **C)**, Quantitative analysis of positive MOMA-2, MCP-1 and IL-6 staining; **(D, E** and **F)**, Western blot analysis of protein expression of MCP-1 and IL-6 in 3 groups of mice and quantitative analysis. ^*^P <0.05 vs. Ang II group; #*P* <0.05 vs. si-NC group. **(G, H** and **I)**, Western blot analysis of protein expression of MCP-1 and IL-6 in macrophages and quantitative analysis.^*^P <0.05 vs. Ang II group; #*P* <0.05 vs. si-NC group.

Experimental data support that AngII infusion could upregulate macrophage infiltration and proinflammatory cytokine expression in aortic tissue of ApoE^−/−^ mice [13, 14]. We examined the *in vivo* presence of macrophages in suprarenal aortic tissue by immunohistochemistry. Macrophage infiltration was lower in si-RELMβ group than that in Ang II and si-NC groups (*P* <0.05, Figure 2A and 2B). In addition, immunohistochemical staining showed significantly downregulated protein expression of inflammatory cytokines such as monocyte chemoattractant protein 1 (MCP-1) and interleukin 6 (IL-6) in suprarenal aortic tissues in si-RELMβ group than those in Ang II and si-NC groups (*P* <0.05, Figure 2A and 2C). Protein expression assessed by western blot showed similar results among the 3 groups (*P* <0.05, Figure 2D-2F). These results suggest that the inflammatory progress in response to Ang II was blunted with RELMβ inhibition.

We next assessed whether RELMβ deficiency affected the *in vitro* levels of inflammatory cytokines in macrophages. Ang II could promote and augment the activation of inflammation in macrophages [14]. RELMβ-deficient macrophages showed less protein expression of MCP-1 and IL-6 than control and si-NC macrophages (*P* <0.05, Figure 2G-2I).

We added recombinant RELMβ (rRELMβ) into the culture to mimic the role of exogenous RELMβ in inflammatory response and found obvious production of proinflammatory cytokines (MCP-1 and IL-6) (*P* <0.05, Figure 4A and 4B). Thus, we confirmed that RELMβ promoted the secretion of proinflammatory cytokines.

**Figure 3:**
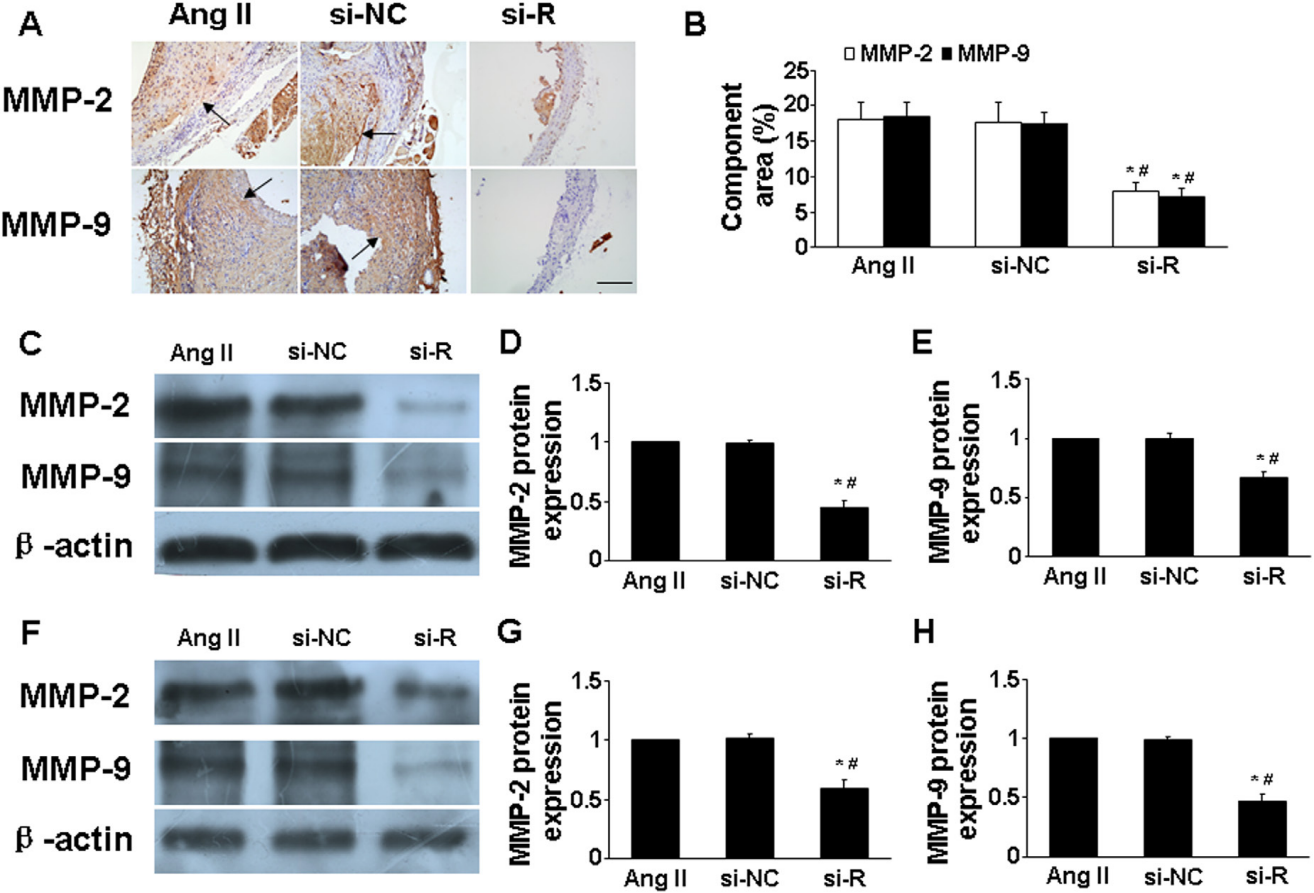
Effect of RELMβ gene deletion on the expression of MMP-2 and MMP-9 *in vivo* and *in vitro* **(A)**, Representative immunohistochemical staining of MMP-2 and MMP-9 in 3 groups of mice; Scale bar: 100 μm. **(B)**, Quantitative analysis of results in A; **(C, D** and **E)**, Western blot analysis of protein expression of MMP-2 and MMP-9 in 3 groups of mice and quantitative analysis. ^*^P <0.05 vs. Ang II group; #*P* <0.05 vs. si-NC group. **(F, G** and **H)**, Western blot analysis of protein expression of MMP-2 and MMP-9 in macrophages and quantitative analysis. ^*^P <0.05 vs. Ang II group; #*P* <0.05 vs. si-NC group.

**Figure 4:**
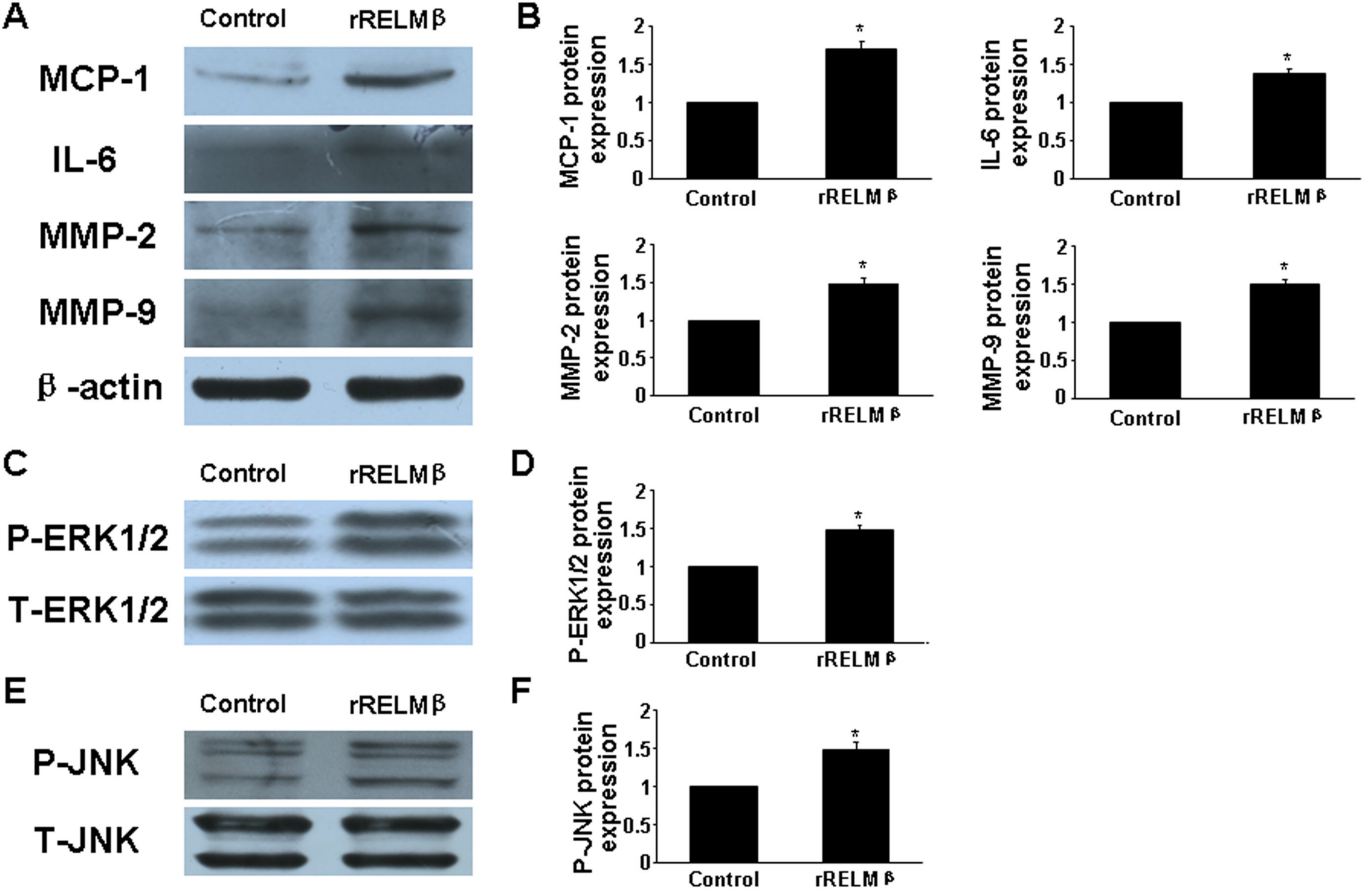
Effect of rRELMβ stimulation on cytokine expression and MAPKs signaling pathway *in vitro* **(A)**, Western blot analysis of protein expression of MCP-1, IL-6, MMP-2 and MMP-9; **(B)**, Quantitative analysis of results in A; **(C** and **D)**, Western blot analysis of P-ERK1/2 and total ERK1/2 (T-ERK1/2) and quantitative analysis of P-ERK1/2 normalized to T-ERK1/2 protein expression; **(E** and **F)**, Western blot analysis of P-JNK and total JNK (T-JNK) and quantitative analysis of P-JNK normalized to T-JNK protein expression. ^*^P <0.05 vs. control group.

### RELMβ knockdown attenuated matrix metalloproteinase 2 (MMP-2) and MMP-9 expression

Ang II treatment greatly increased MMP-2 and MMP-9 expression in aortic tissues in ApoE^-/-^ mice [13]. In the present study, si-RELMβ treatment downregulated the protein expression of MMP-2 and MMP-9 in aorta assessed by immunohistochemistry and western blot as compared with Ang II and si-NC treatment (*P* <0.05, Figure. 3A-3E). In macrophages, the high levels of MMP-2 and MMP-9 induced by Ang II were significantly decreased in si-RELMβ group, when compared to the Ang II and si-NC groups (*P* <0.05, Figure. 3F-3H). On the contrary, rRELMβ-stimulated macrophages showed greater MMP-2 and MMP-9 protein expression than controls (*P* <0.05, Figure 4A and 4B).

### RELMβ was involved in activating the mitogen-activated protein kinases (MAPKs) pathway

**Figure 5:**
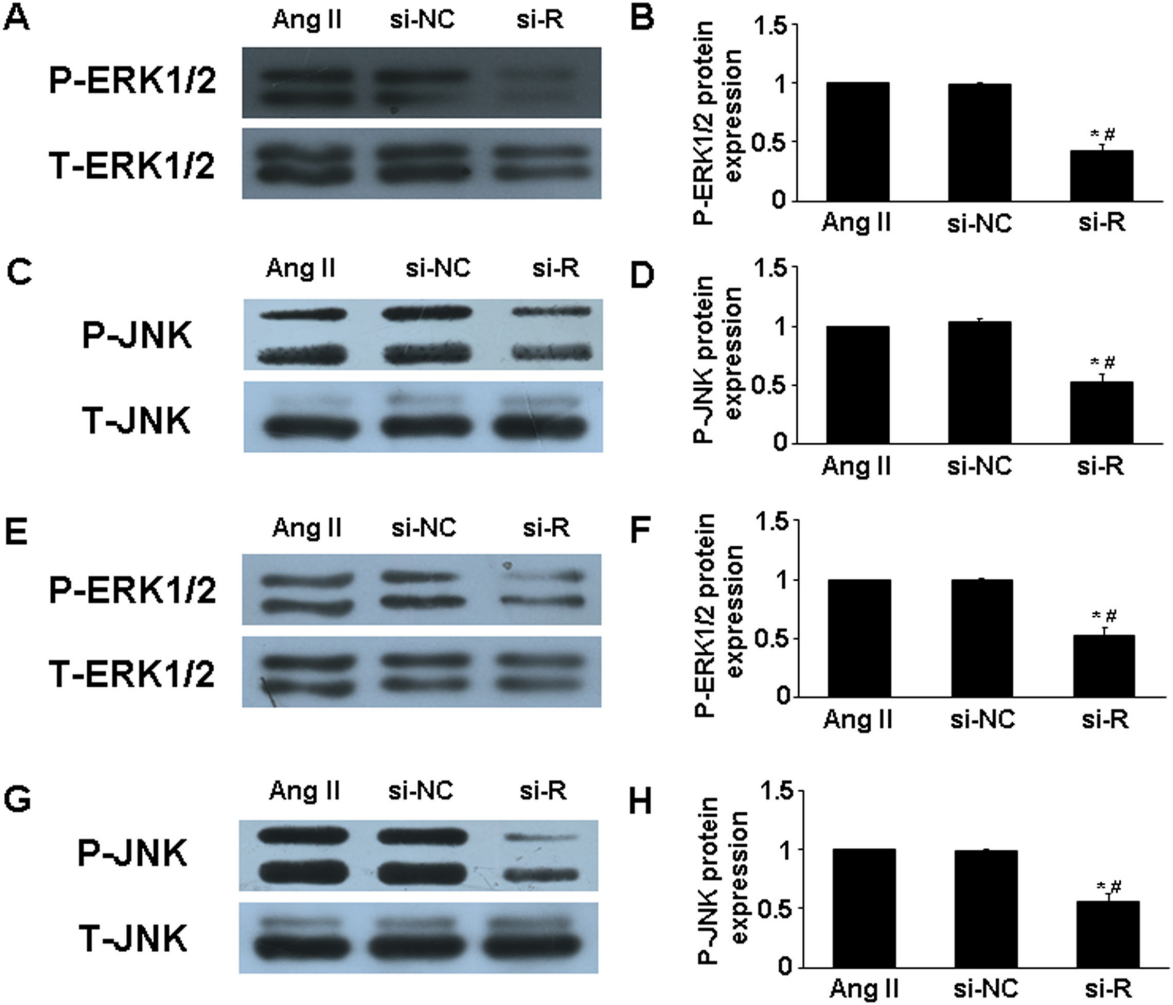
Effect of RELMβ gene deletion on MAPKs signaling pathway *in vivo* and *in vitro* **(A** and **B)**, Western blot analysis of P-ERK1/2 and T-ERK1/2 in 3 groups of mice and quantitative analysis; **(C** and **D)**, Western blot analysis of P-JNK and T-JNK in 3 groups of mice and quantitative analysis; ^*^P <0.05 vs. Ang II group; #*P* <0.05 vs. si-NC group. (**E** and **F**), Western blot analysis of P-ERK1/2 and T-ERK1/2 in macrophages and quantitative analysis; **(G** and **H)**, Western blot analysis of P-JNK and T-JNK in macrophages and quantitative analysis. ^*^P <0.05 vs. Ang II group; #*P* <0.05 vs. si-NC group.

MAPKs (ie, extracellular signal-regulated kinase 1/2 [ERK1/2] and c-Jun N-terminal kinase [JNK]) participate in the pathogenesis of AAA, and Ang II induced their phosphorylation [16]. To further examine the intracellular mechanism of RELMβ in AAA progression, we assessed the role of the MAPKs signaling pathway. The phosphorylation of ERK1/2 (p-ERK1/2) and JNK (p-JNK) in suprarenal aortic segments was lower in si-RELMβ group than that in Ang II and si-NC group (*P* <0.05, Figure. 5A-5D), while there was no difference between Ang II and si-NC treatment. Similar to the *in vivo* study, in cultured macrophages, the expression of p-ERK1/2 and p-JNK was significantly decreased in si-RELMβ group than that in Ang II and si-NC groups (*P* <0.05, Figure. 5E-5H).

**Figure 6:**
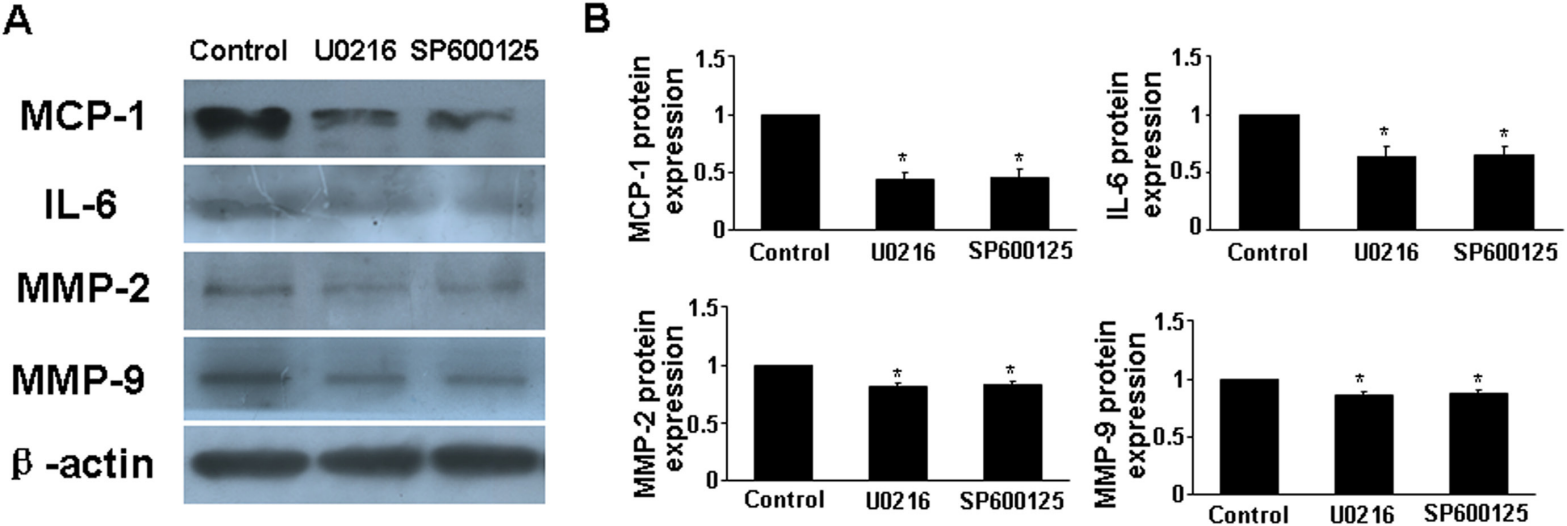
Effect of MAPKs signaling pathway on cytokine expression after rRELMβ stimulation **(A)**, Macrophages were pretreated with the inhibitors of ERK1/2 and JNK, then stimulated with rRELMβ. Western blot analysis of protein expression of MCP-1, IL-6, MMP-2 and MMP-9; **(B)**, Quantitative analysis of results in A.^*^P <0.05 vs. control group.

To determine whether RELMβ promoted the activity of MAPKs, we measured the protein expression of p-ERK1/2 and p-JNK in response to RELMβ in macrophages. P-ERK1/2 and p-JNK expression were increased in rRELMβ-stimulated macrophages as compared with controls (*P* <0.05, Figure. 4C-4F). Next, we further assessed whether MAPKs pathway activation was involved in upregulating the proinflammatory cytokines, MMP-2 and MMP-9 mediated by RELMβ. Pretreatment with specific inhibitors of ERK1/2 and JNK partly abolished the upregulated MCP-1, IL-6, MMP-2 and MMP-9 induced by rRELMβ (*P* <0.05, Figure. 6). Therefore, RELMβ mediated the pathogenesis of AAA, which may involve the activation of the ERK1/2 and JNK pathway.

## DISCUSSION

AAA always results in progressive dilation, even abrupt rupture of the aorta, and represents high mortality. Chronic vascular inflammation, excessive extracellular matrix (ECM) degradation and intense oxidative stress imply pathophysiological progression in human AAA [17, 18]. However, the precise pathogenesis of AAA is still unclear. RELMβ, a secretory protein homologous to resistin, increases insulin resistance [19]. Our primary study suggested an internal association between RELMβ and AAA formation. Here, we further elucidated the effect of RELMβ in aneurysm formation with Ang II-induced animal models of AAA and provided complementary data to demonstrate that RELMβ knockdown protected against AAA formation. The inherent mechanism included inhibiting inflammatory cell accumulation and suppressing proinflammatory cytokines expression and MMP-2 and MMP-9 production in the vascular wall.

*In vivo* experiment, we used loss-of-function approaches by lentivirus delivery into mice for RELMβ silencing to address a precise role of RELMβ in AAA formation. The siRNA was used to block the potency of RELMβ *in vivo* and in macrophages. As expected, our experimental findings supported the transfection efficiency of RELMβ siRNA.

Ang II is known a vasoconstrictor propertie, and chronic infusion of Ang II could lead to increased blood pressure in ApoE^−/−^ mice. However, AAA formation is independent of increased blood pressure [20]. In this study, RELMβ gene defeciency had no effect on blood pressure in response to Ang II in mice. In addition, experimental findings suggest that AAA development is not associated with plasma lipid levels [21]. In our animal experiments, RELMβ gene silencing did not affect serum lipid levels, which was consistent with previous study [6].

An inflammatory mechanism is implicated in the progression of AAA, and vascular inflammation has been found an important initiating factor and feature in human AAA [14, 22]. Aneurysmal segments are characterized by an inflammatory response with increased accumulation of proinflammatory cells and local proinflammatory cytokine production in arterial wall tissues [14, 22]. These cells and various proinflammatory cytokines represent a complex inflammatory network that contributes to tissue destruction and results in aortic rupture [23]. Macrophages are major proinflammatory cells and a critical part of the inflammatory progress; they secrete cathepsins, degrade wall elastin and destroy the structural integrity of the vascular wall. The activation and recruitment of macrophages is the prominent initiating factor in the progression of aneurysm [1, 14]. Ang II infusion promoted the accumulation of macrophages and vascular inflammatory response in adventitia of ApoE^−/−^ mice [14]. Prior study revealed that RELMβ, a proinflammatory molecule in the intestine, promoted the intestinal inflammatory response, whereas RELMβ^−/−^ mice showed reduced inflammatory cell accumulation and proinflammatory cytokine expression in the colon and resisted intestinal inflammation [24, 25]. In addition, RELMβ affects the M1/M2 balance and the induction of macrophages transforming into foam cells and stimulating inflammatory cytokine production; blocking the effect of RELMβ could blunt the inflammatory response [6, 26]. Therefore, RELMβ may be a target for controlling inflammatory response.

We observed macrophage and proinflammatory cytokine expression in aortic segments to assess the role of RELMβ and found decreased macrophage infiltration and inflammatory mediator expression in aortic tissues of RELMβ-deficient mice associated with blunted local inflammation. *in vitro*, RELMβ-deficient macrophages significantly alleviated the activation of proinflammatory mediators, whereas rRELMβ-stimulated macrophages accelerated the production of proinflammatory cytokines. Therefore, RELMβ may promote the activation of macrophages, accelerate the inflammatory status and trigger the inflammatory signaling cascade both in aortic tissues and macrophages. Inflammation has mechanistic significance in the pathogenesis of AAA and blunting the inflammatory response may be beneficial for the prevention of AAA formation and progression. Therefore, reduced RELMβ levels may benefit the suppression of AAA formation.

ECM is the structural framework of the arterial wall and provides a structural support to vascular health. Excess ECM degradation contributes to aortic expansion, mediates the degradation and destruction of the aortic wall and is believed to play a central role in the pathogenesis of AAA [27, 28]. AAA formation requires matrix degradation, and human AAA is characterized by excess degradation and remodeling of ECM [29]. Much attention has been given to ECM degradation in AAA development. MMPs, mainly secreted by resident macrophages, could lead to ECM degradation and destruction of the vascular wall [1]. Aberrant expression of MMPs is thought to be indispensable for AAA formation [30]. Ang II induces MMPs secretion and activation, and increased MMPs expression promotes a weakening of the structural matrix of the wall and internal elastic lamina disruption. Ang II-induced AAA formation is associated with augmented MMPs expression, whereas inhibition of MMPs may impair aneurysm formation in animal models [30-32]. MMP-2 and MMP-9 are the predominant members of the MMP family in the AAA wall; their expression is enhanced in human aneurysmal tissues and they have an essential role in the generation of AAA [30, 33, 34]. Increased activity of MMP-2 and MMP-9 promotes the degradation of elastin and collagen, weakens the artery wall and thus facilitates AAA formation [17]. AAA formation and rupture mostly depends on MMP-2 and MMP-9 [27]. In *in vivo* study, the expansion rate of AAA was reduced by inhibiting MMP-2 and MMP-9 [30, 33]. Thus, therapeutic agents that focus on inhibiting MMP-2 and MMP-9 expression may benefit AAA formation and progression. In this study, RELMβ deficiency greatly decreased MMP-2 and MMP-9 expression in response to Ang II both *in vivo* in mouse aortas and *in vitro* in macrophages, to perhaps protect against aortic matrix destruction and tissue integrity loss. However, in the study by Zheng et al, overexpression of RELMβ by transfecting RELMβ vector into gastric cancer cells inhibited MMP-2 and MMP-9 expression [35]. Perhaps RELMβ plays different roles in different pathological conditions.

The MAPKs family has a fundamental role in cell signaling and is involved in insulin resistance. As members of the MAPKs family, ERK1/2 and JNK are associated with inflammatory signaling and MMPs activity and are implicated in the pathogenesis of AAA formation; inhibition of ERK1/2 and JNK prevented AAA progression in a mouse model [15, 36]. In animal experiments, Ang II stimulation promoted the phosphorylation of ERK1/2 and JNK [16]. In primary cultured hepatocytes, RELMβ could activate ERK1/2 and JNK [37]. Here, ERK1/2 and JNK activation was significantly attenuated in RELMβ-deficient mice and macrophages. Concomitantly, rRELMβ promoted the activation of ERK1/2 and JNK *in vitro*, whereas the upregulated proinflammatory cytokine expression induced by rRELMβ was reversed by pretreatment with ERK1/2 and JNK inhibitors. Thus, activation of ERK1/2 and JNK may be involved in the mechanism of RELMβ in AAA progression, which sheds light on the molecular mechanism of RELMβ-implicated signaling in the progression of AAA.

In conclusion, deletion of RELMβ protected against AAA formation in an experimental animal model, which suggests an important role of RELMβ in the pathogenesis of AAA. RELMβ may be a potential molecular target for novel anti-aneurysm agents and a possible strategy for AAA treatment.

## MATERIALS AND METHODS

### Preparation of lentiviral vectors and target screening of siRNA

Recombinant lentivirus was prepared and small interfering RNA (siRNA) technology was used to block the RELMβ axis (si-RELMβ). The siRNA sequence of RELMβ was 5’-GGAGAGTGAATCTGCTCTTAG-3’. The siRNA for the negative control (si-NC) was a targeted scrambled siRNA sequence with unknown homology to mammal genes: 5’-TTCTCCGAACGTGTCACGT-3’. Recombinant lentivirus contained the siRNA for RELMβ or negative control (Shanghai Genechem Co., China).

### Experimental animals

In total, 60 3-month-old male ApoE^−/−^ mice were housed under specific pathogen-free conditions and fed a Western-type diet (0.25% cholesterol and 15% cocoa butter). All animals had free access to food or water and were maintained on a 12-h light/dark cycle.

After anaesthesia, mice were implanted with a minipump for continuous subcutaneous infusion of saline (n=15) or Ang II (1000 ng/kg/min, n=45) for 4 weeks. Saline-infused mice were negative controls. Before Ang II treatment, Ang II-treated mice were randomly allocated to 3 groups: Ang II, si-NC and si-RELMβ (n=15 each group), for intravenous injection of saline, lentivirus containing si-NC (2×10 [7] TU per mouse) and lentivirus containing si-RELMβ (2×10 [7] TU per mouse), respectively. Four weeks later, all animals underwent euthanasia, blood samples were collected via cardiac puncture, and aortic tissues were removed for analysis.

### Ethics statement

All protocols used in the study were approved by the Animal Ethics Committee of Shandong University. The methods complied with the approved guidelines of the Animal Management Rules of the Chinese Ministry of Health (Document No. 55,2001).

### Blood pressure measurement

During the experiments, SBP was measured in conscious mice by the tail-cuff system (Softron BP-98A. Tokyo). SBP was averaged from 3 consecutive readings.

### Lipid profile

Blood samples were prepared to determine serum lipid profile (total cholesterol and triglycerides) by enzymatic assay.

### Histology and morphology

Immediately after mice were euthanized, the abdominal aorta was dissected and fixed in 4% paraformaldehyde overnight. The maximal outer diameter of the suprarenal aorta was measured by computerized morphometry. AAA was defined as ≥ 50% dilation of the external diameter as compared with the normal suprarenal aorta in saline-infused mice [10]. A scoring system was used to evaluate aneurysm severity [11]: type 0, normal suprarenal aorta; type I, dilated lumen without thrombus; type II, remodeled tissue with little intraluminal thrombus; type III, a conspicuous bulbous form of type II containing thrombus; type IV, multiple overlapped aneurysms containing thrombus. After aortas were harvested, 5-μm-thick sections were prepared for staining with hematoxylin and eosin (H&E), Verhoeff-Van Gieson (VVG) and immunohistochemical staining with antibodies to identify macrophages (MOMA-2, diluted 1:100, AbD Serotec, UK), MCP-1 (1:100), IL-6 (1:100), MMP-2 (1:100) and MMP-9 (1:100, all Abcam, UK). Analysis of slides involved use of Image Pro Plus 6.0 (Media Cybernetics, USA).

### Cell culture

RAW mouse macrophages (Manassas, VA, USA) were cultured in medium in 6-well plates and used at passage 4. Macrophages were grown to near confluence and were randomly divided into 3 groups: Ang II, si-NC and si-RELMβ, receiving no treatment or transfection with lentivirus (MOI=40): si-NC-LV and si-RELMβ-LV, respectively. After transfection with or without lentivirus for 72 h, cells were stimulated with Ang II (1 μM) for another 24 h.

In another experiment, cultured macrophages were stimulated with or without rRELMβ (PeproTech, 1 μg/ml) for 24 h. To evaluate the mediator role of MAPKs, including ERK1/2 and JNK, macrophages were pretreated with the inhibitor of ERK1/2 (U0216, 10 μmol/L) or JNK (SP600125, 20 μmol/L) (all Cell Signaling Technology, USA) for 1 h, followed by a 24 h-incubation with rRELMβ (1 μg/ml). Then, cells of all groups were harvested for further analysis.

### Real-time PCR analysis

The suprarenal aortic segments of mice were separated from fat and connective tissues. Total RNA was isolated from suprarenal aortas or macrophages of each group by using Trizol reagent (Invitrogen). Relevant cDNA was generated following the manufacturer’s protocol, and mRNA expression was assayed by use of SYBR Green Supermix (Bio-Rad, CA, USA) underwent real-time PCR. Each sample was detected in triplicate and the relative mRNA expression analysis involved the 2^-∆∆CT^ method. The customized primers were for mouse RELMβ: forward 5’-GCTCTTCCCTTTCCTTCTCCAA-3’, reverse 5’-AACACAGTGTAGGCTTCATGCTGTA-3’; and mouse β-actin: forward 5’-CACTGTGCCCATCTACGA-3’, reverse 5’-GTAGTCTGTCAGGTC CCG-3’.

### Western blot analysis

Protein was isolated from the suprarenal aortic segments of mice or lysed macrophages and separated by SDS-PAGE, then electrotransferred onto a polyvinylidene difluoride (PVDF) membrane (Amersham Biosciences, NJ, USA), blocked in 5% skim milk for 2 h at room temperature, and incubated with primary antibodies for β-actin (1:1000, Cell Signaling Technology, USA); MCP-1 (1:500), IL-6 (1:500), MMP-2 (1:500), and MMP-9 (1:1000, all Abcam); and ERK1/2 (1:1000), P-ERK1/2(1:1000), JNK (1:1000) and P-JNK (1:1000, both Cell Signaling Technology). After being washed in TBS-T 3 times for 10 min, membranes were incubated with horseradish peroxidase-conjugated secondary antibodies for 2 h and visualized by use of chemiluminescent HRP substrate (Millipore, USA).

### Statistical analysis

Statistical analysis involved use of SPSS v13.0 (SPSS Inc, Chicago, IL, USA). Categorical variables are presented as number and percentage and continuous variables as mean±SD. Student 2-tail *t*-tests and one-way ANOVA with least significant difference post-hoc analysis were used for comparison as appropriate and differences were considered statistically significant at *P* <0.05.

## SUPPLEMENTARY MATERIALS FIGURE AND TABLES


