# Supplementary material for: Deletion of resistin-like molecule-beta attenuates angiotensin II-induced abdominal aortic aneurysm
Source: Oncotarget. 2017 Oct 24;8(61):104171–81. doi: 10.18632/oncotarget.22042 (PMC5732796; doi:10.18632/oncotarget.22042)
Supplement: Supplementary file 1 [file oncotarget-08-104171-s001.pdf]

Deletion of resistin-like molecule-beta attenuates angiotensin II-induced abdominal aortic aneurysm

SUPPLEMENTARY MATERIALS

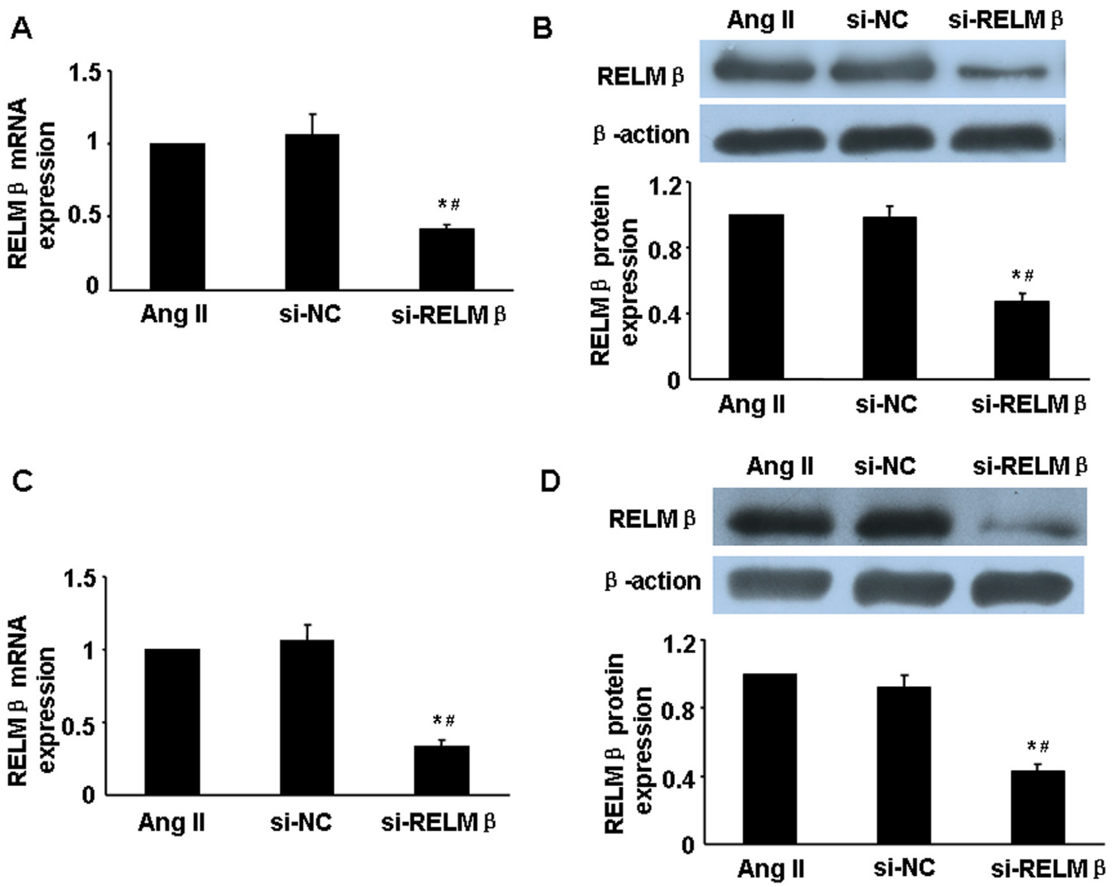

Supplementary Figure 1: Transfection efficiency of RELMβ siRNA *in vivo* and *in vitro*. (A) RT-PCR analysis of RELMβ mRNA expression in 3 groups of mice; **(B)** Western blot analysis of RELMβ protein expression in 3 groups of mice and quantitative analysis; *P <0.05 vs. Ang II group, #P <0.05 vs. si-NC group. **(C)** RT-PCR analysis of RELMβ mRNA expression in macrophages; **(D)** Western blot analysis of RELMβ protein expression in macrophages and quantitative analysis. *P <0.05 vs. Ang II group; #P<0.05 vs. si-NC group.

Supplementary Table 1: Systolic blood pressure (SBP) in Ang II-infused mice

SBP (mmHg)	Ang II	si-NC	si-RELMβ
0 weeks	102±6	100±7	101±5
4 weeks	152±5*	155±7*	157±6*

*P <0.05 vs. SBP (0 weeks) in the same group.

Supplementary Table 2: Serum lipid levels in Ang II-infused mice

Parameters	Ang II	si-NC	si-RELMβ
TC (mmol/L)	26.17±3.79	24.52±3.78	25.14±3.64
TG (mmol/L)	1.42±0.30	1.39±0.32	1.40±0.36

TC, total cholesterol; TG, triglycerides.
